# A survey of the full-length transcriptome of *Gracilariopsis lemaneiformis* using single-molecule long-read sequencing

**DOI:** 10.1186/s12870-022-03992-0

**Published:** 2022-12-19

**Authors:** Xiaojiao Chen, Yue Yao Tang, Haodong Yin, Xue Sun, Xiaoqian Zhang, Nianjun Xu

**Affiliations:** Key Laboratory of Marine Biotechnology of Zhejiang Province, School of Marine Sciences, Ningbo University, Ningbo, 315211 Zhejiang China

**Keywords:** *Gracilariopsis lemaneiformis*, SMRT-seq, Posttranscriptional processing, Alternative splicing, Alternative polyadenylation

## Abstract

**Background:**

Posttranscriptional processing of precursor mRNAs contributes to transcriptome and protein diversity and gene regulatory mechanisms in eukaryotes. However, this posttranscriptional mechanism has not been studied in the marine macroalgae *Gracilariopsis lemaneiformis,* which is the most cultivated red seaweed species in China.

**Results:**

In the present study, third-generation sequencing (Pacific Biosciences single-molecule real-time long-read sequencing, SMRT-Seq) was used to sequence the full-length transcriptome of *G. lemaneiformis* to identify alternatively spliced transcripts and alternative polyadenylation (APA) sites in this species. RNAs were isolated from *G. lemaneiformis* under various treatments including abiotic stresses and exogenous phytohormones, and then equally pooled for SMRT-Seq. In summary, 346,544 full-length nonchimeric reads were generated, from which 13,630 unique full-length transcripts were obtained in *G. lemaneiformis*. Compared with the known splicing events in the gene models, more than 3000 new alternative splicing (AS) events were identified in the SMRT-Seq reads. Additionally, 810 genes were found to have poly (A) sites and 91 microRNAs (miRNAs), 961 long noncoding RNAs and 1721 novel genes were identified in *G. lemaneiformis*. Moreover, validation experiments showed that abiotic stresses and phytohormones could induce some specific AS events, especially intron retain isoforms, cause some alterations to the relative ratios of transcripts annotated to the same gene, and generate novel 3′ ends because of differential APA. The growth of *G. lemaneiformis* was inhibited by Cu stress, while this inhibition was alleviated by ACC treatment. RNA-Seq analysis further revealed that 211 differential alternative splicing (DAS) events and 142 DAS events was obtained in CK vs Cu and Cu vs Cu + ACC, respectively, suggesting that AS of functional genes could be regulated by Cu stress and ACC. Compared with Cu stress, the expression of transcripts with DAS events mainly involved in the carbon fixation in photosynthetic organisms and oxidative phosphorylation pathway was upregulated in Cu + ACC treatment, revealing that ACC alleviated the growth inhibition by Cu stress by increasing carbon fixation and oxidative phosphorylation.

**Conclusions:**

Our results provide the first comprehensive picture of the full-length transcriptome and posttranscriptional mechanism in red macroalgae, including transcripts that appeared in the presence of common abiotic stresses and phytohormones, which will improve the gene annotations of *Gracilariopsis* and contribute to the study of gene regulation in this important cultivated seaweed.

**Supplementary Information:**

The online version contains supplementary material available at 10.1186/s12870-022-03992-0.

## Introduction

*Gracilariopsis/Gracilaria lemaneiformis* (*G. lemaneiformis*) is the most important cultivated red alga due to its high yield, it is commercially valuable for agar and polysaccharide production [1, 2]. In China, the cultivated area of *G. lemaneiformis* is second only to that of *Saccharina japonica*, reaching 10,459 ha and its yield reached 36,8967 tons dry weight in 2020 (2021 China Fishery Statistical Yearbook). *G. lemaneiformis* has been used as a high-quality feed for abalone, shrimp, snapper, and other economically important fish species [3]. In addition, *G. lemaneiformis* can absorb a large amount of N and P substances, improve water quality and provide the environmental benefits [4]. Second-generation transcriptome sequencing (RNA-Seq) is one of the fundamental genetic technologies used to study gene expression, and it can reveal complex biological processes in *G. lemaneiformis* and other species. However, the short read length of RNA-Seq can make accurately obtaining complete transcripts difficult, due to repeat regions and genome complexity. Although the genome sequences of *G. lemaneiformis* and *G. chorda* have been released [5, 6], they cannot be used as the reference genome of RNA-Seq because of the low comparison rates between the CDS sequences in the genome and those from RNA-Seq. These results suggest that the transcriptome of *G. lemaneiformis* is complex and that precursor mRNAs (pre-mRNAs) may undergo numerous posttranscriptional processes, such as alternative splicing (AS) and alternative polyadenylation (APA). This phenomenon has been widely confirmed in mammals, whose genomes are far less than what is needed for required. The ‘missing’ information is largely provided by AS, which synthesizes multiple different functional mRNAs [7], and APA, which generates mRNAs with alternative 3′ ends [8].

Currently, single-molecule real-time long-read (SMRT) sequencing from Pacific Biosciences (PacBio) allows direct sequencing of complete RNA molecules and yields full-length sequences with an average read length of 3000 bp and a maximum length of 15 kb [9]. SMRT sequencing has great advantages for whole transcriptome profiling and determining the structure of mRNAs, which increases the discovery of new genes and the detection of AS events and other types of posttranscriptional processes such as APA, as well as long noncoding RNAs (lncRNAs) and microRNAs (miRNAs) [10, 11]. Simultaneously, full-length transcriptome profiling improves the assembly and annotation of the genome. Furthermore, many studies have shown that abiotic stresses have a significant effect on the AS and APA of pre-mRNAs in plants [12–14]. AS responds to acute stresses, and the transcriptional patterns of AS genes are significantly enhanced under stress conditions [10, 15].

In recent years, the cultivation of *G. lemaneiformis* has developed rapidly along the southern coasts of China, especially in Fujian and Guangdong Province. However, the high temperature in the summertime along the southern coasts is a key ecological factor that damages living *G. lemaneiformis*. The optimum growth temperature of cultivated *G. lemaneiformis* is between 16 °C and 26 °C [16]. Temperatures exceeding 26 °C can disrupt metabolic processes and hinder the normal growth and development of *G. lemaneiformis*, which leads to death and ultimately yield reduction [17]. Heavy metal contaminations, such as cuprum (Cu), lead, cadmium and zinc, are some of the most common pollutants in coastal environments and have become a serious threat to seaweed cultivation in China [18, 19]. Among these heavy metals, Cu participates in the electron transport chain and lipid peroxidation [20] but harms the growth and photosynthesis of *G. lemaneiformis* at concentrations as low as 1.0 μM [21, 22].

Phytohormones were reported to be involved in the defense against abiotic stress. Abscisic acid (ABA) can be induced by heat stress in plants [23] and can reduce the damage caused by heat stress in *G. lemaneiformis* [24]. The content of 1-aminocyclopropane-1-carboxylic acid (ACC), the direct precursor of ethylene, increases under heavy metal stress conditions [25]. In the presence of heavy metal stress, ACC promotes antioxidant defense mechanisms, such as upregulating the expression of SOD and CAT genes in plants and algae [26, 27]. Interestingly, it was observed in *Arabidopsis* that phytohormones dramatically alter the AS of pre-mRNA and enhance the complexity of the transcriptome [28].

In the present study, our aim was to obtain as many full-length transcripts as possible; thus, RNA samples of *G. lemaneiformis* were collected from various treatments including abiotic stresses and exogenous phytohormones added under stress conditions and equally pooled for sequencing on the PacBio Sequel platform. Then, posttranscriptional processing of pre-mRNAs, including AS, APA, miRNA and lncRNA, and the identification of novel genes was analyzed. Furthermore, some AS, APA and miRNA events were randomly selected to study the effect of abiotic stresses and phytohormones on their expression. Finally, RNA-Seq was performed in *G. lemaneiformis* thalli under Cu stress, Cu + ACC treatment, and control condition and used to analyze the differential alternative splicing (DAS) events and transcripts.

## Results

### SMRT-Seq data and error correction

**Fig. 1:**
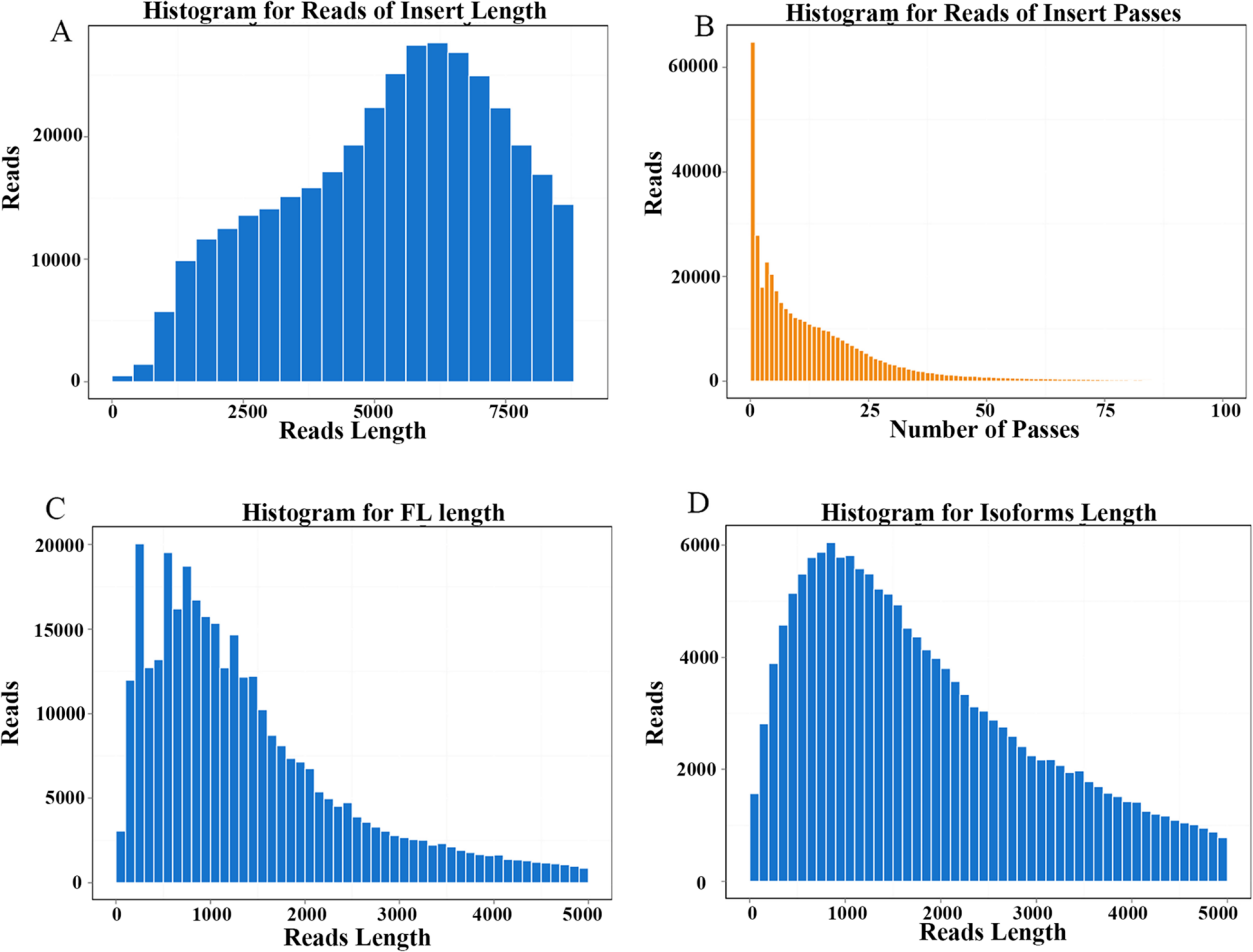
PacBio single-molecule long-read sequencing data output statistics. **A** The full pass of ROI sequence distribution. **B** cDNA library CCS length distribution. **C** Length distribution of full-length nonchimeric reads. **D** Length distribution of consensus isoform

After filtering out low-quality subreads, 34.79 Gb of clean reads remained from SMRT sequencing, and 429,088 of reads of insert (ROIs) with average length of 6136 bp and 14 passes were obtained (Figs. 1A, B). In addition, 346,544 (80.76%) of the ROIs were full-length, nonchimeric (FLNC) transcripts containing 5′ primer, 3′ primer and poly(A) tail. The average read length of the FLNC transcripts was 1602 bp (Fig. 1C). Furthermore, 166,867 final consensus isoform sequences (mean quality = 0.99) with an average length of 2150 bp were obtained (Fig. 1D).

The consensus isoforms were compared to the reference genome, and redundant reads were removed based on their genomic locations; 95.53% of the reads were aligned to the reference genome, and 7623 (70.54%) reference genes (refgenes) had transcript coverage. After removing redundant reads, a total of 13,630 unique transcripts with an average length of 1570 bp were generated for subsequent analysis. Among these unique transcripts, 10,461 (76.7%) were intronless, 9589 (70.4%) overlapped with a single annotated protein-coding gene, 1665 (12.2%) overlapped two or more adjacent annotated genes and 2375 (17.4%) did not overlap with any annotated genes. In a previous study [29], the alignment rate between CDSs within genomes and those from RNA-Seq was approximately 40%, while the alignment rate between unique transcripts from SMRT-Seq and those from RNA-Seq was over 85% (Fig. S1). This result suggested that unique transcript data obtained from SMRT-Seq can be used as a reference for RNA-Seq. Furthermore, 18 fusion genes which are far enough on the genome were detected (Table S2).

### Splice isoforms and AS event analysis

**Fig. 2:**
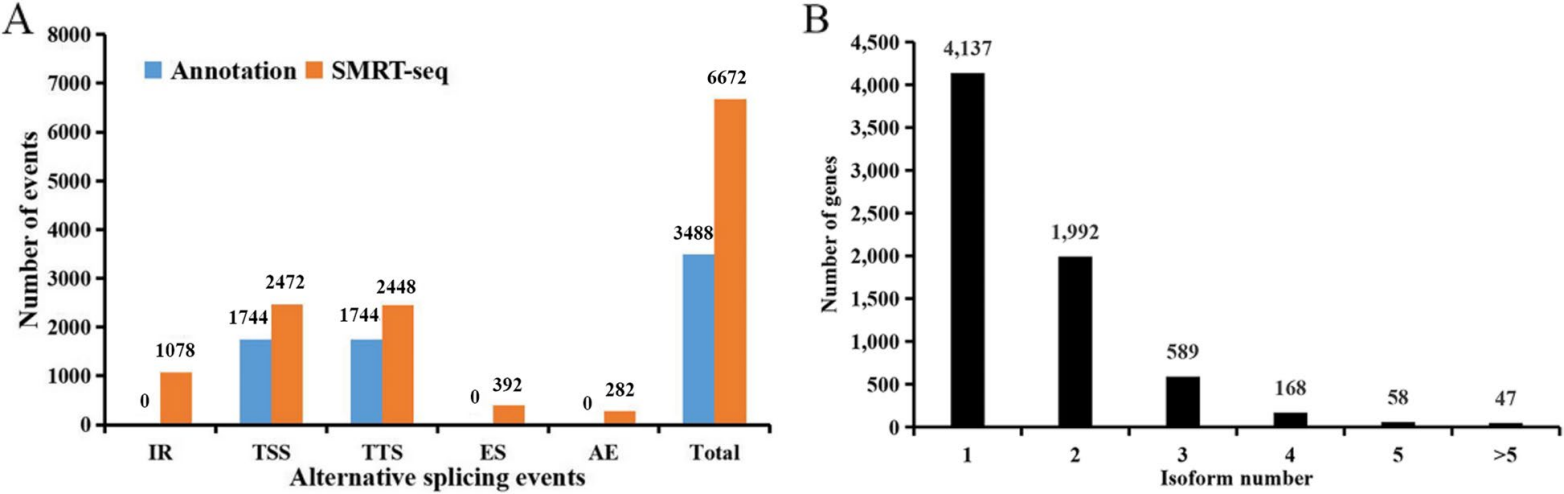
Alternative splicing and splice isoform analysis with SMRT-Seq reads. **A** The total number of AS events in predicted genes according to SMRT-Seq data compared with the annotated gene models. Annotation, AS events in genes expressed in gene models; SMRT-Seq, AS events in genes expressed in Iso-Seq reads. IR, intron retention; TSS, alternative transcription start site; TTS, alternative transcription termination site; ES, exon skipping; AE, alternative exon ends; and Total, all AS events. **B** Distribution of genes that produce one or more splice isoforms in SMRT-Seq data of *G. lemaneiformis*

In SMRT-Seq reads, 6672 AS events were predicted, whereas only 3488 AS events were present in the gene models (Fig. 2A). The types of AS events were shown in Fig. S2. The identification of more than 3000 new AS events included 1078 intron retention (IR), 728 alternative transcription start site (TSS), 704 alternative transcription start site (TTS), 392 exon skipping (ES) and 282 alternative exon ends (AE) events in the SMRT-Seq reads. This result suggested that numerous AS events were not annotated in the published genome of *Gracilariopsis*. Then we summarized the number of isoforms from the SMRT-Seq reads against the corresponding genes. In 4137 genes (69.9%), only one transcript was detected, while 2854 (40.5%) genes possessed more than one transcript, for a total of 7052 isoforms (Fig. 2B). In addition, 5 or more splice isoforms were discovered in 105 genes (Fig. 2B).

**Fig. 3:**
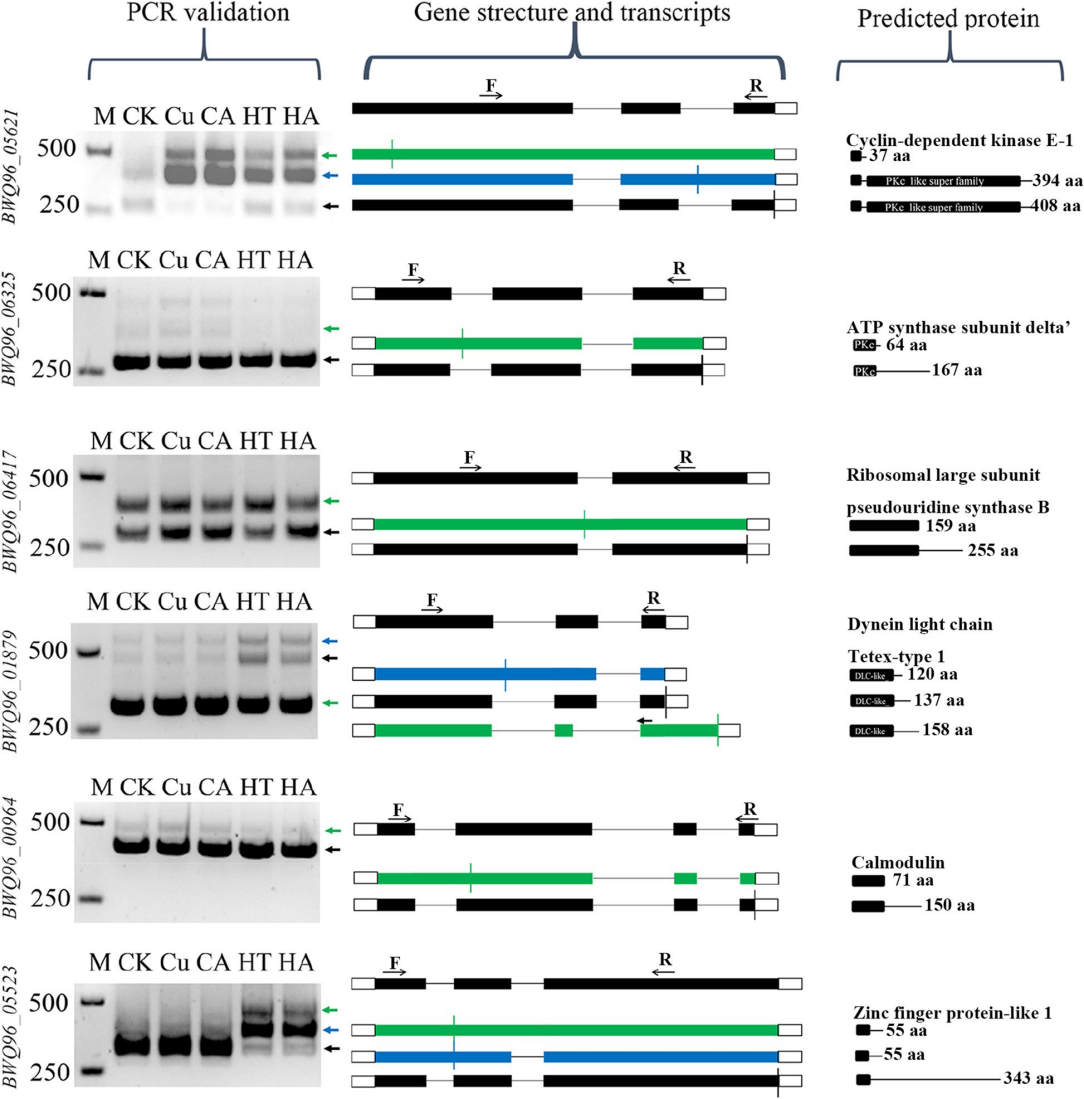
PCR validation of AS events identified in SMRT-Seq data. cDNAs from control (CK), and treated (Cu^2+^ (Cu), Cu^2+^ combined with ACC (CA), 33 °C (HT) and 33 °C combined with ABA (HA)) samples were used for RT–PCR. Exons are represented by filled boxes, introns are represented by lines, and 3′ and 5′ UTRs are represented by open boxes. The annotated isoforms are shown in black. The novel isoforms from SMRT-Seq reads and sequencing of PCR products are colored and indicated by arrows. The right panel shows the predicted protein of each isoform and putative domains identified by MEME. The location of the predicted stop codon in the transcripts is represented by a vertical line. The gene ID is shown at the left in each panel. M, lane with DNA size markers. F, forward primer and R, reverse primer

The size of the fragments in the gel banding pattern was observed to be consistent with the AS transcripts recognized in the SMRT-Seq data (Fig. 3). Then the fragments were purified, cloned, and sequenced. The sequences of the validated transcripts and their corresponding genes are shown in Fig. S3. We observed that some splice isoforms were differentially expressed under stress, which altered the ratio of AS isoforms. For instance, one transcript from *BWQ96_05621* and *BWQ96_05523* was only induced in the stress-treated samples and the ratio of spliced isoforms from *BWQ96_05621*, *BWQ96_01879* and *BWQ96_05523* varied between the control and treated samples (Fig. 3). Moreover, the expression and ratio of AS isoforms differed in response to Cu and heat stress. The expression of two IR isoforms of *BWQ96_05621* induced by Cu stress was higher than that induced by heat stress, whereas the expression of isoforms of *BWQ96_01879* and *BWQ96_05523* showed opposite results (Fig. 3). In addition, phytohormones resulted in changes in the expression level of *BWQ96_06417* isoforms (Fig. 3). These results showed that abiotic stresses and phytohormones could remarkably alter the ratio of AS isoforms.

### Alternative polyadenylation detection

**Fig. 4:**
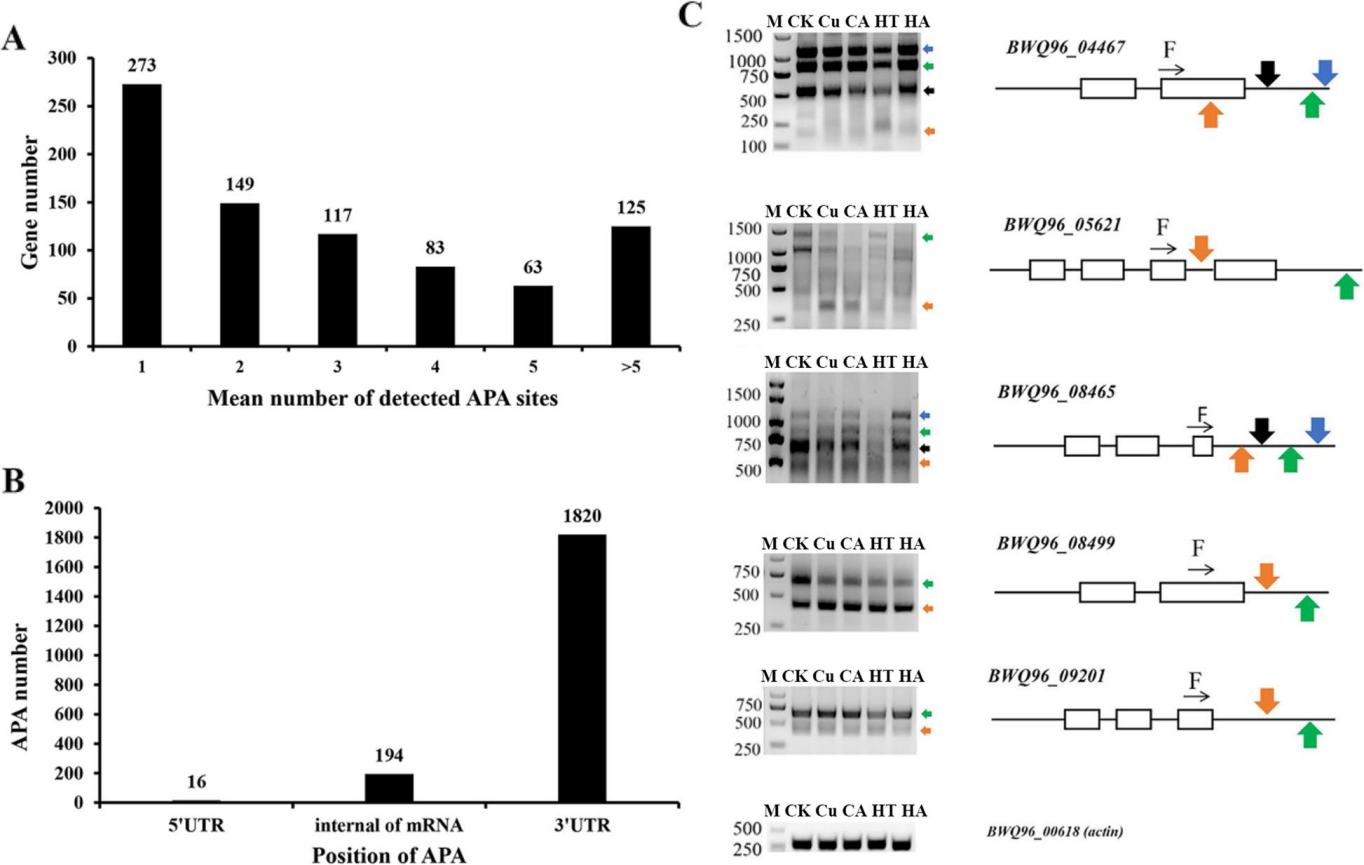
Alternative polyadenylation analysis. **A** Distribution of the number of poly (A) sites per gene. **B** The statistics of APA locations within genes. **C** Validation of polyadenylation sites by RT–PCR. PCR was performed using a 3′ RACE reverse primer and gene-specific forward (F) primer. The colored arrows indicate the locations of APAs and point to the corresponding PCR product. Exons are represented by open boxes. Equal amounts of cDNA in the samples were verified using *actin* as an internal control

In *G. lemaneiformis*, 810 genes have at least one supported poly(A) site, resulting in 2030 unique poly(A) sites (Figs. 4A, B). The types of APA events and detailed poly(A) sites are shown in Fig. S2 and Table S3, respectively. Among the poly(A) sites, most APA sites (89.7%) were located in the 3’UTR of mRNA, additionally, several internal APA sites (9.6%) were found, while the least frequent type was 5′ UTR APA site (0.79%) (Fig. 4B). In addition, novel 3′ ends were generated due to various APA sites in the gene models.

Five genes were randomly selected for validation of APA events, and gel electrophoresis results of the predicted the fragments in the gel based on SMRT-Seq prediction fragments were confirmed by sequencing (Fig. 4C). The expression level and ratio of APA events were changed under stresses, as seen for APA events of *BWQ96_05621*, *BWQ96_08465* and *BWQ96_08499*, while phytohormones only altered the expression of APAs events of *BWQ96_05621* (Fig. 4C)*.* These results indicated that APA was regulated by stresses and phytohormones in *G.lemaneiformis*.

### MiRNA and lncRNA identification

**Fig. 5:**
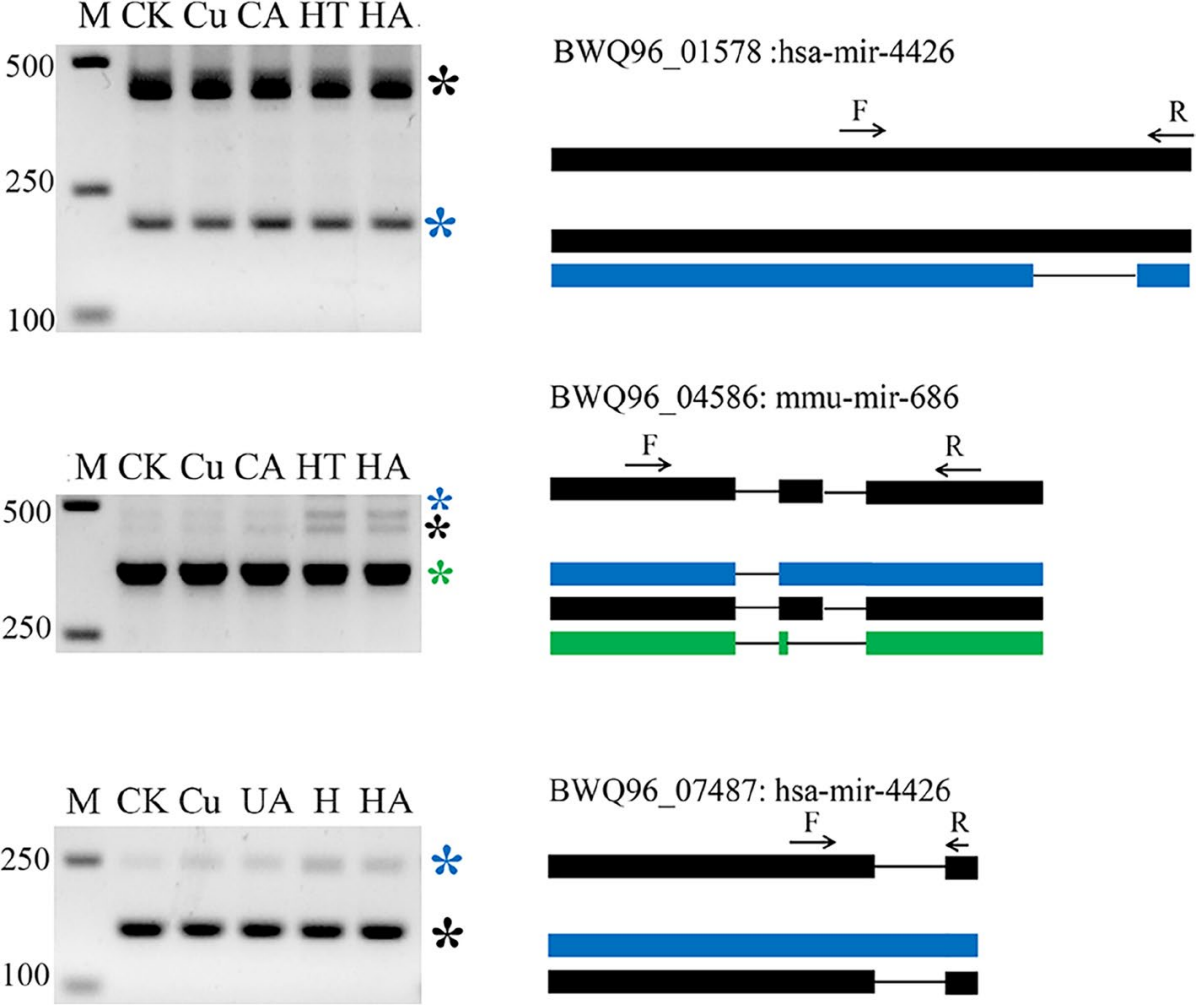
Validation of the expression and splicing of miRNAs by RT–PCR. Exons are represented by filled boxes and introns by lines. F, forward primer; R, reverse primer

The unique transcripts were searched against published miRNA sequences curated at miRBase, and 91 miRNAs including 81 new transcripts were obtained (Table S4). Of these miRNAs, 40, 28 and 23 miRNAs were aligned to animal, microorganism, and plant miRNAs, respectively (Table S4). Seven genes containing miRNAs had multiple transcripts and exhibited 26 AS events (Table S4). The expression of two isoforms of the miRNA (BWQ96_04586: mmu-mir-686) was induced by heat stress, while that of two other miRNAs was not affected by stresses or phytohormones (Fig. 5). In addition, 961 lncRNAs with a mean length of 375.5 bp were obtained, of which 957 and 463 lncRNAs were new transcripts and novel genes, respectively. These results showed that miRNAs and lncRNAs were mainly composed of new transcripts in *G.lemaneiformis*.

### Novel gene annotation

**Fig. 6:**
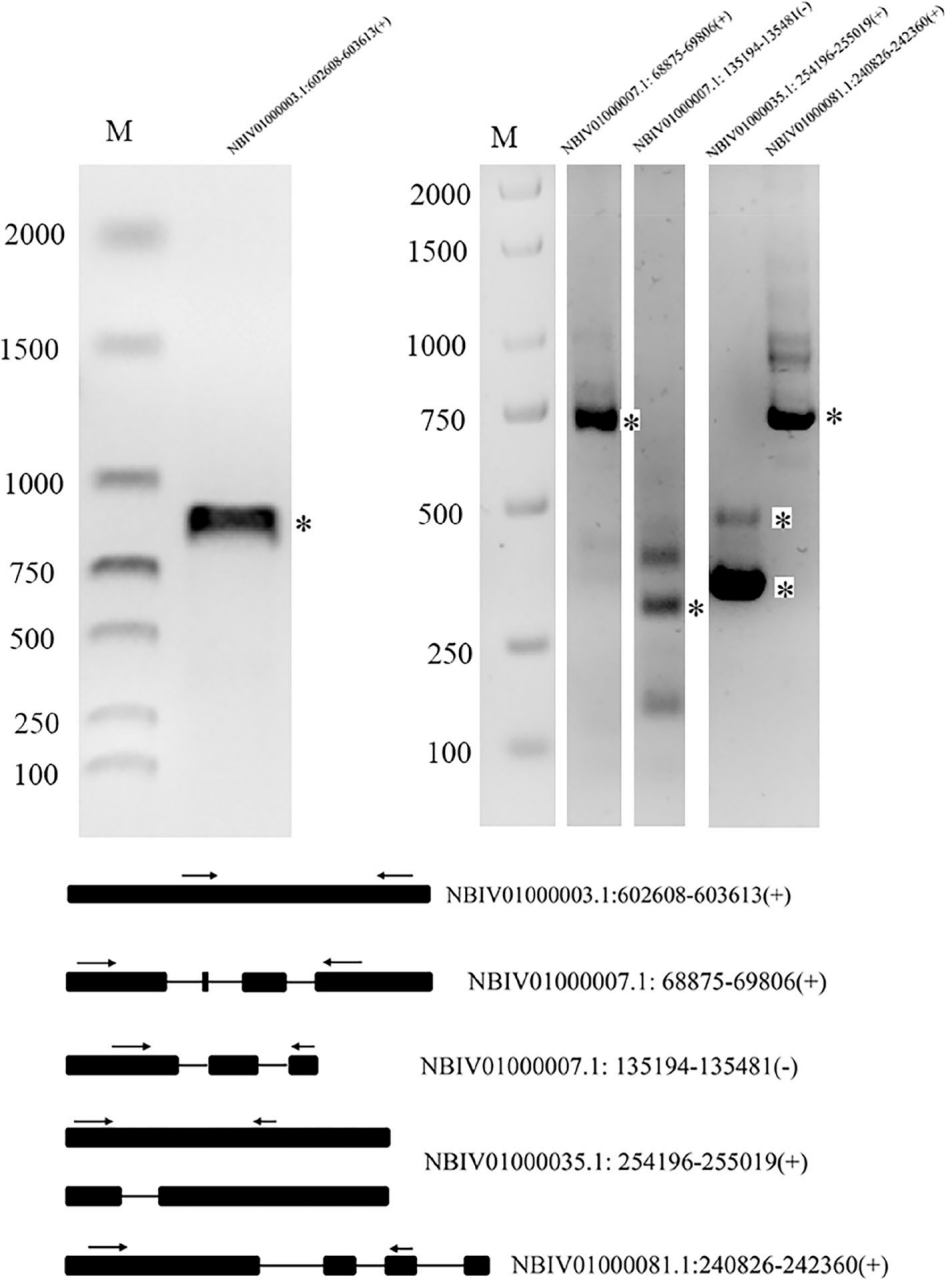
The expression levels and splicing events of novel genes were verified by RT–PCR. The arrows indicate the primer sets

In the SMRT-Seq results, 1721 unique transcript clusters without any overlapping annotated genes were identified as novel genes. These predicted novel genes were annotated in seven databases, including 365 in Gene Ontology (GO), 266 in Kyoto Encyclopedia of Genes and Genomes (KEGG), 278 in Eukaryotic orthologous group (KOG), 491 in Protein Families, 215 in SwissProt, 143 in Nt, and 615 in Non-Redundant Protein Database (Table S5). Furthermore, 18 novel genes were annotated to Transcription factors (TFs), including the IIIA, bHLH and Myb superfamilies (Table S6). Approximately 44.7% of the novel genes were annotated in at least one database (Table S5). As shown in Fig. 6, the expression and AS events of these novel genes were verified by sequence analysis.

### Analysis of DAS transcripts and corresponding pathways

**Fig. 7:**
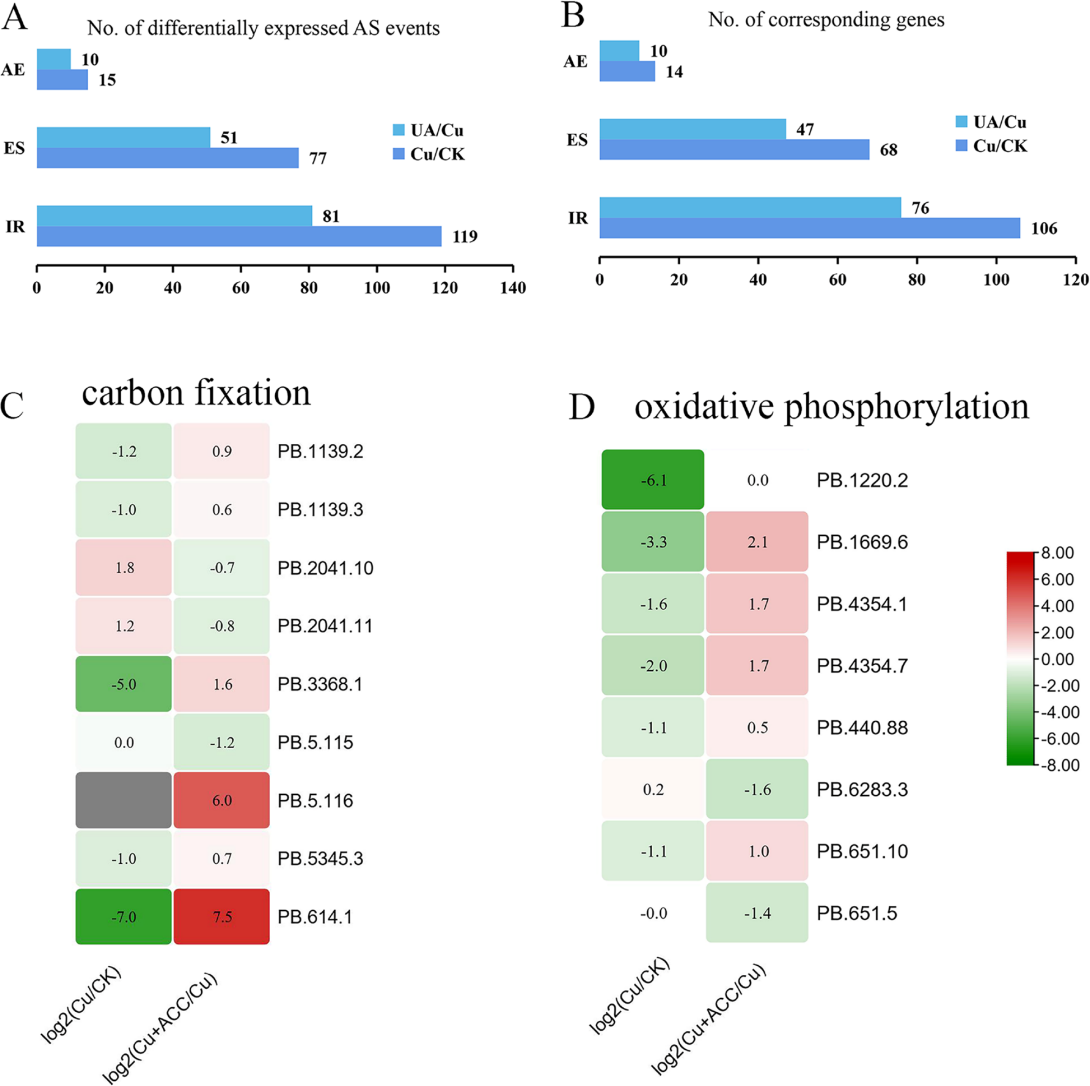
Differential alternative splicing (DAS) and related pathways identified in *G. lemaneiformis*. **A**, **B** The total number of each type of DAS event (**A**) and corresponding genes (**B**) from RNA-Seq data in response to Cu and Cu + ACC, respectively. **C**, **D** Expression of transcripts involved in carbon fixation in photosynthetic organisms (**C**) and oxidative phosphorylation (**D**) pathway in response to Cu and Cu + ACC, respectively

The effect of Cu and the addition of ACC under Cu stress on the biomass of *G. lemaneiformis* was shown in Fig. S4. Cu significantly inhibited relative growth rate (RGR) of *G. lemaneiformis* by 51.0%, while RGR in the addition of ACC under Cu stress (Cu + ACC) samples was 43.0% higher than that in Cu-stress samples (Fig. S4), suggesting that ACC could alleviate the inhibited growth by Cu in *G. lemaneiformis*. To examine the change of stress- or hormone-responsive AS events, RNA-Seq was conducted in *G. lemaneiformis* thalli treated with CK, Cu, and Cu + ACC. Clean reads of RNA-seq were mapped to the 17,963 transcripts containing genomic-annotated transcripts and SMRT-Seq-identified isoforms. We identified 1926 differentially expressed transcripts (DETs) and 1359 DETs in CK vs Cu and Cu vs Cu + ACC, respectively. Then, 1489, 1583 and 1573 AS events were identified under CK, Cu, and Cu + ACC treatments, respectively. Among these, a total of 211 DAS events (10.9% of DETs) from 178 gene loci and 142 DAS events (10.4% of DETs) from 133 gene loci was obtained in CK vs Cu and Cu vs Cu + ACC, respectively (Figs. 7A, B).

GO, KEGG enrichment [30] and TFs analysis of the transcripts with DAS events was performed. As a result, the transcripts with DAS events in Cu-treated samples were remarkably annotated to GO terms of ADP binding, glyceraldehyde-3-phosphate dehydrogenase (NAD(P)^+^), carbohydrate metabolic process, oxidoreductase activity, anion channel activity and photosynthesis (Fig. S5). Correspondingly, the KEGG pathway enrichment analysis displayed that the transcripts with DAS events mostly involved in carbon fixation in photosynthetic organisms, glycolysis/gluconeogenesis, and biosynthesis of amino acids (Fig. S5). Compared to Cu stress group, the transcripts with DAS events in Cu + ACC-treated samples were remarkably annotated to GO terms of mitochondrial envelope, organelle membrane, alternative oxidase activity, posttranslational protein targeting to endoplasmic reticulum membrane and ion binding (Fig. S5). The KEGG pathway enrichment analysis showed that the transcripts with DAS events mostly involved in proteasome, oxidative phosphorylation, and carbon fixation in photosynthetic organisms in Cu vs Cu + ACC (Fig. S5). These findings indicated that carbon fixation in photosynthetic organisms and oxidative phosphorylation of DAS may participate in Cu and Cu + ACC treatment in *G. lemaneiformis*. Under Cu stress, the expression of most of the transcripts with DAS events enriched in carbon fixation in photosynthetic organisms and oxidative phosphorylation pathway was down-regulated (Figs. 7C, D), while expression patterns opposite in Cu + ACC vs Cu group (Figs. 7C, D), revealing that ACC alleviated the harm by Cu stress through increasing carbon fixation and oxidative phosphorylation. In addition, 11 DAS events from 9 gene loci was annotated to TFs including MYB, C2H2, bHLH, C2C2-CO-like (Table S7). Meanwhile, Cu stress and ACC treatment altered the ratio of AS isoforms of TFs (Table S8), consistent with the AS validation by RT-PCR. These results suggested that alternative splicing of functional genes can be regulated by Cu stress and ACC.

## Discussions

The transcriptome is the intermediate link between the genetic information of the genome and the biological function of the proteome. Identification of full-length splice transcripts is necessary for understanding the properties of the encoded protein and evaluating the role of splice variants in gene regulation. SMRT sequencing technology provides information on splice variants by producing full-length transcripts [9]. In this study, we obtained several novel findings from SMRT sequencing, as follows: i) 13,630 unique transcripts were obtained and 7623 (70.54%) refgenes had transcript coverage; ii) among the detected refgenes, 2854 genes produced multiple transcripts and approximately 3000 new AS events were predicted from the SMRT-Seq reads; iii) 810 genes were found to have poly(A) site; and iv) 1721 novel genes not previously annotated were identified. Our results showed that posttranscriptional regulation was complex in *Gracilariopsis* and the predicted splicing transcripts and APA sites could be important resources for reannotating the *Gracilariopsis* genome.

AS, which is important for the eukaryotic genetic information flow, enhances transcriptome and subsequent proteomic diversity and is highly pervasive in higher plants [7, 12]. The ratio of AE events, which represent alternative 5′ and 3′ splice sites, within the AS events of *Gracilariopsis* was lower than that of higher plants [7] (Fig. 2A). Our results showed that IR is the predominant mode of AS, which is consistent with most land plants in the PISO database, including *Arabidopsis*, sorghum, and maize [10, 12, 31]. It has been reported that IR events are regulated in the nucleus and may be widespread as storage form of precursor RNA [12]. IR may regulate developmental processes of plants or respond to signals from the environment [12]. RNA-Seq analysis showed that the transcripts with DAS events, affected by Cu and ACC treatment, accounted for approximately 10% of DETs and had biology functions (Fig. 7). Furthermore, in the AS validation results and RNA-Seq analysis, IR of DAS events was main subtype in response to environmental stresses and phytohormones (Figs. 3, Fig. 7).

APA, a widespread posttranscriptional processing, enhances the complexity of the transcriptome by producing mRNAs with alternative 3′ ends [8]. Tandem 3′ UTR APA, including the cleavage of 3’UTRs, is the most widely used form of APA [8], which is consistent with our results (Fig. 4B). 3’UTRs are a major docking platform for miRNAs and RNA-binding proteins. Through alteration of 3’UTRs, APA can regulate multiple aspects of mRNAs, including their stability, localization, and protein translation efficiency [8]. APA sites, which exist inside mRNAs, can change the sequences of transcripts and encoded proteins [8]. In *Arabidopsis*, approximately 15.5% of the APA sites were in the internal regions of mRNAs and a small number of APA sites were located within the 5’UTRs of corresponding genes [32]. Our results suggested that the posttranscriptional regulation pattern of APA events in *G. lemaneiformis* may be consistent with that in higher plants.

MiRNAs, a class of *trans*-acting short (20–24 nucleotides) noncoding RNAs, are the key posttranscriptional regulators of gene expression in eukaryotes [33]. Mature short miRNAs are generated from primary transcripts of miRNAs through two sequential site-specific endonucleolytic events [9]. In this study, we identified 91 miRNAs including 81 new transcripts and 16 novel genes, in *G. lemaneiformis* (Table S4). In plants, miRNAs usually pair with the 3′-UTR of an mRNA, triggering the endonucleotide cleavage of the mRNA and inhibiting the biosynthesis of proteins [34]. However, animal miRNAs may also act on the 5′ UTR and coding regions of mRNAs. The association with 5’UTR sites appears to stimulate translation [35]. Among the predicted miRNAs, 44.0% were aligned to sequences in animals (Table S4)*.* Additionally, in recent years, lncRNAs, which are highly expressed in plants, have been reported to be important regulators of posttranscriptional processing [36]. In the present study, we identified 962 lncRNAs with a mean length of 375.46 bp, of which 99.5% (957) were new transcripts. Thus, new transcripts, which are the major members of miRNAs and lncRNAs, might play an important role in posttranscriptional processes in *G. lemaneiformis*.

In higher plants, alternative splicing profiles are significantly affected by various abiotic stress conditions [37, 38], such as temperature (heat and cold) stress [28, 39], salt stress [15], and drought stress [10, 15]. Many SR genes display AS specific responses and changes to relative ratios of AS types under a given stress [28]. This phenomenon appeared in our study, in which some genes showed AS-specific and ratio alterations under abiotic stresses (Figs. 3, Fig. 7, Table S8). Differential APA and miRNA expression was observed under drought stress in sorghum [10]. Similarly, Cu and heat stresses also caused the differences in APA and miRNA levels in *G. lemaneiformis* (Figs. 4C, Fig. 5). Phytohormones can also alter the AS of pre-mRNAs; for example, ABA, 3-indoleacetic acid and 6-benzyl aminopurine changed the AS of three SR genes in *Arabidopsis* [28]. Our results showed that phytohormones regulated the levels of AS and APA in *G. lemaneiformis* (Figs. 3, 4C, 7).

## Conclusions

Our results provide a comprehensive view of splicing variation in *Gracilariopsis* for the first time, further revealing the mechanism of posttranscriptional processes including AS, APA, miRNA and lncRNA. Based on SMRT-seq, 346,544 FLNC reads were generated, from which 13,630 unique full-length transcripts were obtained and 7623 (70.54%) refgenes had transcript coverage. Among the detected refgenes, 2854 genes produced at least two transcripts, resulting in 7052 isoforms. Compared with AS events in gene models, approximately 3000 new AS events were predicted from the SMRT-Seq reads. In addition, 810 genes were found to have poly(A) sites, resulting in 2030 unique poly(A) sites, and 91 miRNAs, 961 lncRNAs and 1721 novel genes were identified. Furthermore, the validation experiments confirmed the AS isoforms, differential poly (A) sites, miRNAs and novel genes identified in the SMRT-Seq data. RNA-Seq analysis revealed that Cu stresses and ACC had significant effects on the expression of the transcripts with AS events, especially IR isoforms. The predicted AS transcripts and APA sites are an important resource for reannotating this algal genome and greatly enhance transcriptome complexity. The results showed that posttranscriptional splicing and polyadenylation might play vital roles in the adaptation of *G. lemaneiformis* to diverse stresses and its response to phytohormones.

## Methods

### Materials

*G. lemaneiformis* strain 981 was obtained from the Ningde aquaculture base of Fujian, China (26° 65′ N, 119° 66′ E), in October 2021. After being taken to the laboratory, the algae were immediately washed with sterile artificial seawater (salinity 25, 1 L containing 17.53 g NaCl, 0.071 g KBr, 0.5 g KCl, 2.02 mg H_3_BO_4_, 2.92 g Na_2_SO_4_, 2.14 mg NaF, 0.74 g CaCl_2_·2H_2_O, 7.93 g MgCl_2_·6H_2_O, 12.14 mg SrCl·6H_2_O and 0.132 g NaHCO_3_) to remove any sediment and miscellaneous algae. Then, we cultured healthy thalli containing sterile seawater and Provasoli medium for 7 days. *G. lemaneiformis* grew at 23 °C with a photoperiod of 12 L:12D and light intensity of 50 μmol photons m^− 2^ s^− 1^.

In total, 5 samples were obtained after 1 d of treatment, including control (CK), heavy metal (CuCl_2_, 25 μM) (Cu), combined ACC (100 μM) with Cu^2+^ (CA), high temperature (33 °C) (HT) and combined ABA (50 μM) with high temperature (HA). Samples were immediately placed in liquid nitrogen and stored at − 80 °C before RNA extraction.

### RNA preparation, PacBio SMRT library preparation and sequencing

Total RNA was extracted from 0.1 g fresh weight of *G. lemaneiformis* using a RNeasy Plant Mini Kit (Qiagen, Hilden, Germany) with DNase I (Qiagen, Hilden, Germany). The quality and content of RNA were detected using a NanoDrop 2000 spectrophotometer (Agilent Technologies, CA, USA). Finally, 5 samples with the same amount of total RNA were mixed to generate a cDNA library for SMRT sequencing through the PacBio Sequel platform (Biomarker Technologies, Beijing, China).

The library was prepared according to the following steps: mRNA was enriched from the total RNA sample purified by oligo (dT)-attached magnetic beads. The full-length cDNA was produced by a SMARTer™ PCR cDNA Synthesis Kit (Clontech, CA, USA) and cleaved by USER enzyme to enable different transcripts to be linked. Then, cDNA damage and A-tailing were repaired. After cDNA was added to hairpin adaptors at both ends and exonuclease digestion was performed, the SMRTbell library was formed and used for multi-throughput SMRT-Seq.

### Pacific biosciences full length read processing

The SMRT-Seq data were analyzed through ROIs, full-length transcript classification, clustering and polishing to create consensus reads. FLNC transcripts were identified by the poly (A) tail and the 5′/3′ cDNA adaptors and applied for subsequent analysis. The official Isoseq3 toolkit program ‘cluster’ (https://github.com/PacificBiosciences/IsoSeq3) was used to integrate the full-length reads into consensus sequences, followed by error correction with the program ‘polish’, the final consensus isoforms were retained.

The final consensus isoforms were aligned with the reference genome of *G. chorda* using Genomic Mapping and Alignment Program (GMAP) software [40] with default parameters (−t 24 -n 0) due to the lack of a gff file for the *G. lemaneiformis* genome. Then, the consensus isoforms were deduplicated based on the locations of the transcripts in the genome with the official program tofu_collapse. The fusion genes were analyzed using the fusion_finder.py program from the official TOFU 2.2.3 analysis toolkit.

### Alternative splicing and APA site analysis

AS events were analyzed and quantified using ASprofile (http://ccb.jhu.edu/software/ASprofile/). ASprofile compares the assembly results from StingTie with the reference genome, whose results can be used for extraction, quantification, and comparison. The types of AS events included ES, IR, AE, TSS and TTS (Figs. S2 A-E).

The detected APA sites of each gene were analyzed through TAPIS 1.2.1 software [10] based on the GTF file for gene annotations. The APA types were divided into 5’UTR APA, internal APA and 3′ UTR APA according to location on the gene (Fig. S2 F).

### MiRNA and lncRNA prediction

BLASTn analysis was performed using all miRNAs from miRbase to identify putative miRNA transcripts. LncRNAs were predicted using LGC (https://ngdc.cncb.ac.cn/lgc/calculator) by comparison with protein coding RNAs depending on their functional relationships [41].

### Novel gene prediction

Based on the intronic location on the reference genome, the reads were merged into unique genes. Novel genes were defined as those that did not overlap with any annotated genes. The exons and introns of novel genes were predicted using sequences aligned to the genome through Spidey (http://www.ncbi.nlm.nih.gov/spidey/).

### Reverse transcription (RT) -PCR experiment

For cDNA synthesis, 2.5 μg of total RNA was used to obtain cDNA with a PrimeScript II 1st Strand cDNA Synthesis Kit (TaKaRa, Dalian, China). For PCR validation of AS, miRNA and novel genes, 1 μl of the diluted cDNA (1:5) was used in a reaction volume of 10 μl using Neo KOD-Plus (Toyobo, Japan). Six genes, which annotated only one transcript but showed more than one isoform according to SMRT-Seq reads, were chosen to validate AS events. Primers with appropriate regions of these genes were designed for separation AS isoforms by fragment size (Table S1) and carried on reverse transcription (RT)-PCR using RNA from the samples of control and the other four treatments. Specific primers of miRNA and novel genes were also listed in Table S1. For APA validation, the 3′ rapid amplification of cDNA ends (3′ RACE) strategy by the 3′-Full RACE Core Set (TaKaRa, Dalian, China) was applied for the synthesis of cDNA and PCR tests. cDNA was obtained with adaptor primers, and PCR was performed with 3′ RACE primer and gene-specific forward primers (Table S1). *Actin* (*BWQ96_00618*) was used as the internal reference gene.

The cloned DNA fragments were excised from the gel by PCR and purified with a gel extraction kit (Tiangen, Beijing, China). The purified fragments were ligated into the pMD 19-T vector (TaKaRa, Dalian, China) and sequenced for verification. Full uncropped images of all gels used to prepare Figs. 3 and S3, 4C, 5, 6 are shown in Fig. S6, S7,  S8 and S9, respectively.

### RNA-Seq and differential alternative splicing transcripts analysis

RNeasy Plant Mini Kit (Qiagen, Hilden, Germany) was used to extract total RNA from *G. lemaneiformis* after 1 d of control (CK), Cu stress (Cu) and combined Cu with ACC (Cu + ACC) treatment, respectively. Each treatment contains three biological replicates. RNA-seq were performed on BGISEQ-500 by BGI (Shenzhen, China). The reference transcripts were obtained through merging the annotation file from the reference genome and the newly identified isoform annotation file by Iso-Seq using cuffmerge, and extracted the full-length cDNA sequences, which contain transcripts annotated by the reference genome and novel isoform transcripts detected by Iso-Seq. After removing the low-quality reads, the clean data of RNA-Seq were mapped to reference transcripts by Bowtie2 and the transcript abundance in each isoform was normalized to fragments per million reads (FPKM) by Expectation Maximization (RSEM) tool.

Due to the high error rate in the 5′ and 3′ ends of isoforms detected by SMRT-Seq, the TSS and TTS types were excluded for DAS analysis. The transcripts and alternative splicing transcripts with |log2 ^[fold-change (FC)]^ | ≥1 and adjusted *P* value (Q value) ≤0.001 was defined as the DETs and DAS transcripts, respectively. All DAS transcripts were analyzed to GO, KEGG enrichment [30] and TFs database.

## Supplementary Information

**Additional file 1: Figure S1.** The alignment rate between unique transcripts from SMRT-Seq and sequences from RNA-Seq. **Figure S2.** Alternative splicing (AS) (A-E) and alternative polyadenylation (APA) (F) event types. (A) Exon skipping (ES); (B) retention of introns (IR); (C) alternative exon ends (AE); (D) alternative transcription start site (TSS); and (E) alternative transcription termination site (TTS). AS features are in red. (F) APA event types. **Figure S3.** Sequences of the AS transcripts and their corresponding genes. **Figure S4.** The enriched GO categories and KEGG pathways of the transcripts with DAS events under Cu and Cu + ACC conditions. **Figure S5.** Uncropped images PCR of validation of AS events used to prepare Fig. 3. **Figure S6.** Uncropped images PCR of validation of APA events used to prepare Fig. 4C. **Figure S7.** Uncropped images PCR of validation of miRNAs used to prepare Fig. 5.

**Additional file 2: Table S1.** Sequences of primers used for validation of splicing, alternative polyadenylation sites, predicted miRNAs and novel genes. **Table S2.** Fusion genes. **Table S3.** polyA summary. **Table S4.** miRNA analysis. **Table S5.** Novel genes annotated across seven databases. **Table S6.** Transcription factors present in novel genes. **Table S7.** The transcripts with DAS events of TFs in response to Cu and Cu + ACC in *G.lemaneiformis*. The number in each cell is the log2 ^fold change^ (log2^FC^). **Table S8.** The expression quantity and AS ratio of TFs in response to Cu and Cu + ACC in *G.lemaneiformis.*

## Data Availability

The transcriptional data were deposited in the NCBI Sequence Read Archive (BioProject for PacBio SMRT sequencing: PRJNA860347, https://submit.ncbi.nlm.nih.gov/subs/sra/SUB11814316/overview. BioProject for RNA-Seq: PRJNA893895, https://submit.ncbi.nlm.nih.gov/subs/sra/SUB12205331/overview). All data generated or analyzed during this study are included in this published article and its supplementary information files.

## Abbreviations

SMRT-Seq: Aingle-molecule real-time long-read sequencing
APA: Alternative polyadenylation
AS: Alternative splicing
miRNAs: microRNAs
DAS: Differential alternative splicing
RNA-Seq: Second-generation transcriptome sequencing
pre-mRNAs: precursor mRNAs
lncRNAs: long noncoding RNAs
Cu: Cuprum
ABA: Abscisic acid
ACC: 1-aminocyclopropane-1-carboxylic acid
ROIs: Reads of inserts
FLNC: Full-length, nonchimeric
refgenes: Reference genes
IR: Intron retention
TSS: Alternative transcription start site
TTS: Alternative transcription termination site
ES: Exon skipping
AE: Alternative exon ends
GO: Gene Ontology
KEGG: Kyoto Encyclopedia of Genes and Genomes
KOG: Eukaryotic orthologous group
TFs: Transcription factors
DETs: Differentially expressed transcripts
HT: High temperature (33 °C)
GMAP: Genomic Mapping and Alignment Program
RT–PCR: Reverse transcription-PCR
3′ RACE: 3′ rapid amplification of cDNA ends
