# Supplementary material for: A survey of the full-length transcriptome of Gracilariopsis lemaneiformis using single-molecule long-read sequencing
Source: BMC Plant Biol. 2022 Dec 19;22:597. doi: 10.1186/s12870-022-03992-0 (PMC9762032; doi:10.1186/s12870-022-03992-0)
Supplement: Supplementary file 1 — Additional file 1: Figure S1. The alignment rate between unique transcripts from SMRT-Seq and sequences from RNA-Seq. Figure S2. Alternative splicing (AS) (A-E) and alternative polyadenylation (APA) (F) event types. (A) Exon skipping (ES); (B) retention of introns (IR); (C) alternative exon ends (AE); (D) alternative transcription start site (TSS); and (E) alternative transcription termination site (TTS). AS features are in red. (F) APA event types. Figure S3. Sequences of the AS transcripts and their corresponding genes. Figure S4. The enriched GO categories and KEGG pathways of the transcripts with DAS events under Cu and Cu + ACC conditions. Figure S5. Uncropped images PCR of validation of AS events used to prepare Fig. 3. Figure S6. Uncropped images PCR of validation of APA events used to prepare Fig. 4C. Figure S7. Uncropped images PCR of validation of miRNAs used to prepare Fig. 5. [file 12870_2022_3992_MOESM1_ESM.docx]

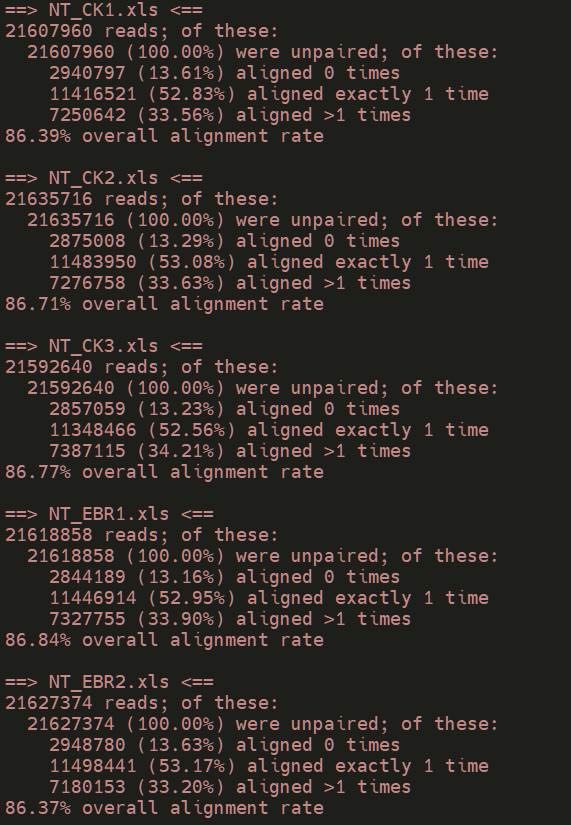


**Figure S1.** The alignment rate between unique transcripts from SMRT-Seq and sequences from RNA-Seq.


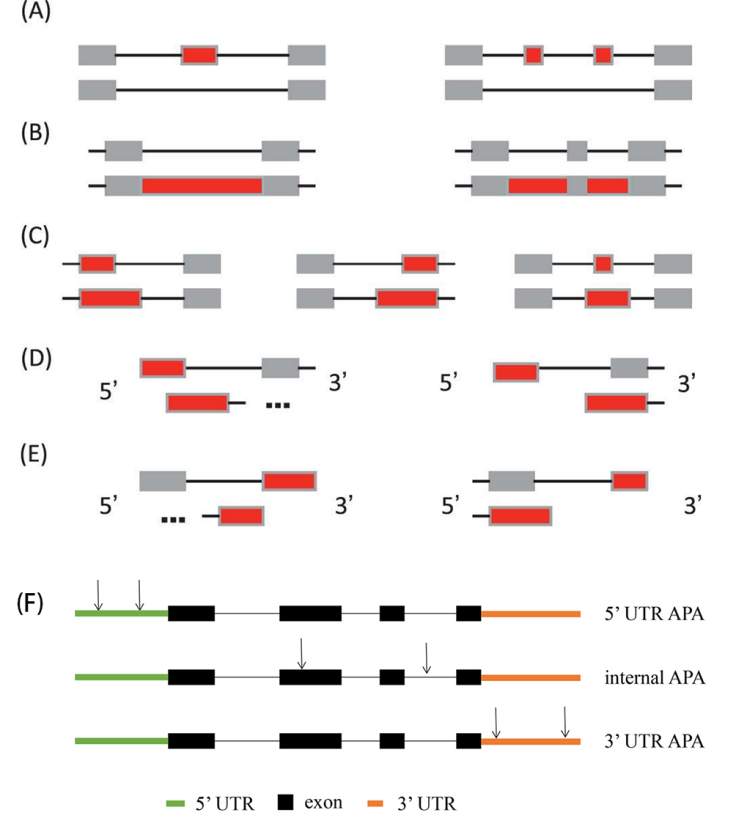


Figure S2. Alternative splicing (AS) (A-E) and alternative polyadenylation (APA) (F) event types.

(A) Exon skipping (ES); (B) retention of introns (IR); (C) alternative exon ends (AE); (D) alternative transcription start site (TSS); and (E) alternative transcription termination site (TTS). AS features are in red. (F) APA event types.


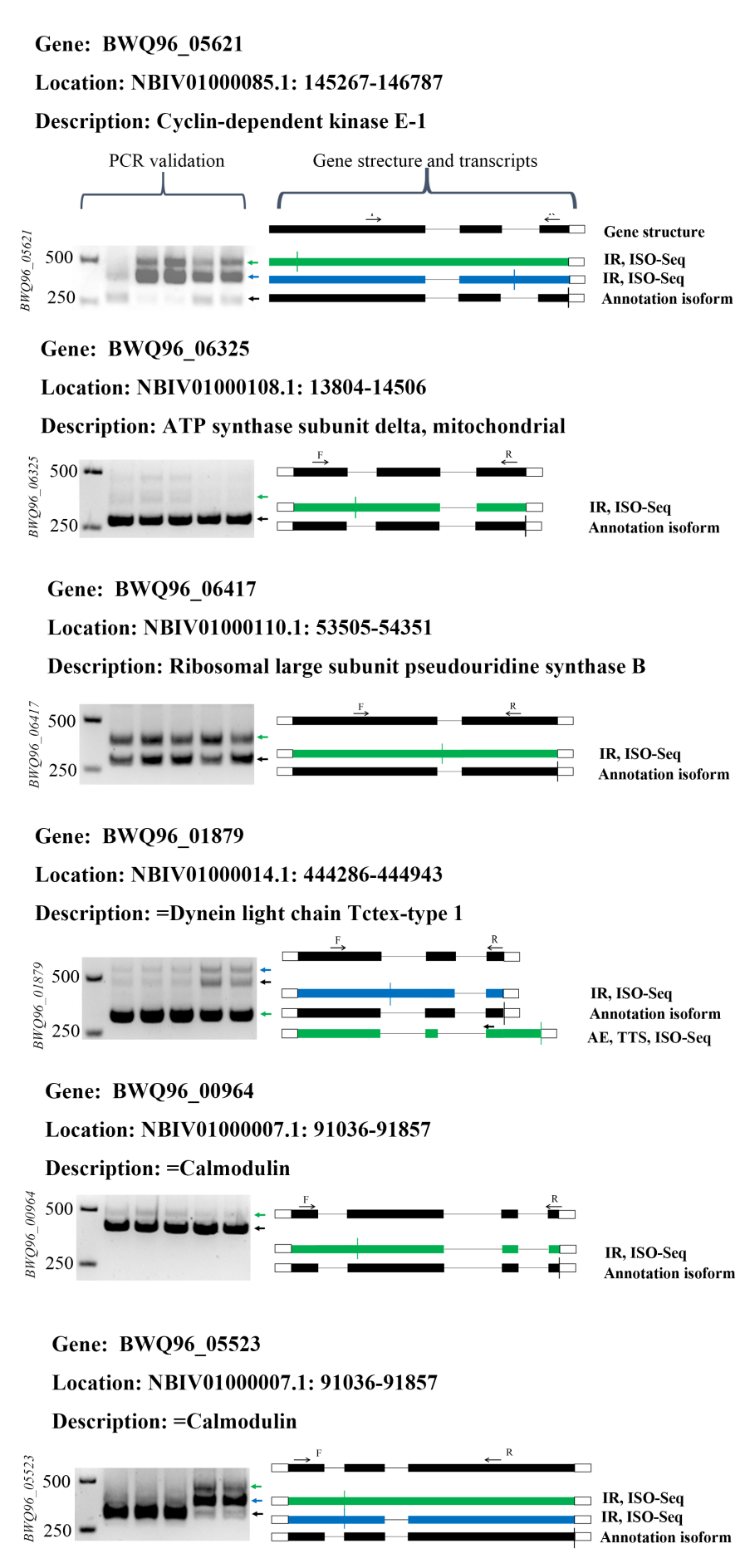


Figure S3. The sequences of the AS transcripts and their corresponding genes.


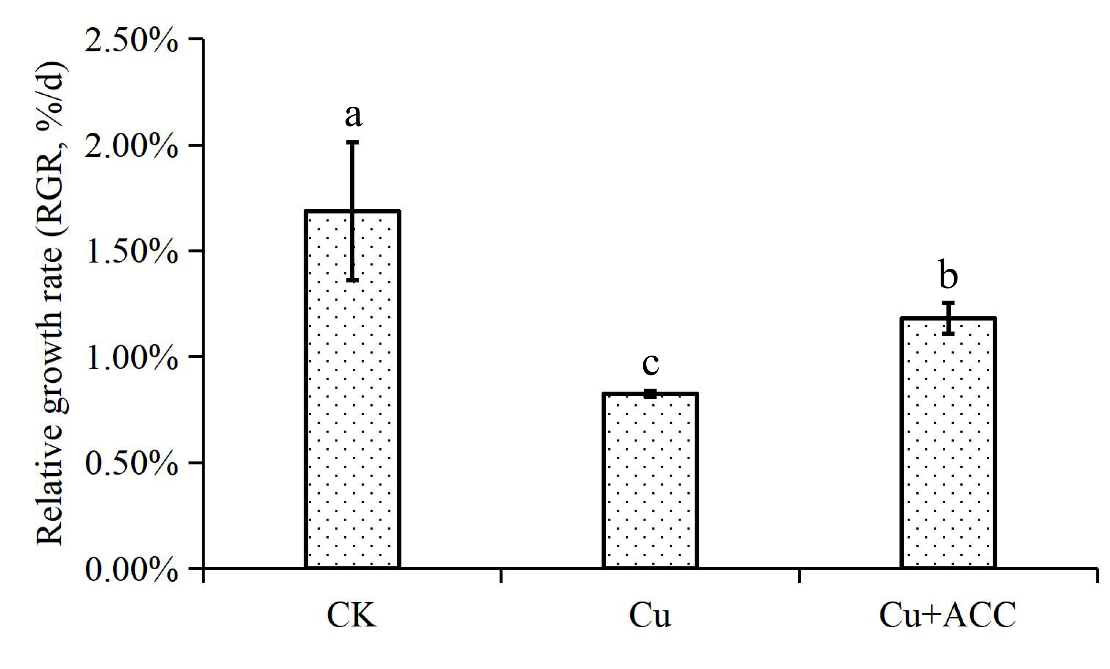


**Figure S4.** Effects of Cu and Cu+ACC on the relative growth rate (RGR) of *G.lemaneiformis.*


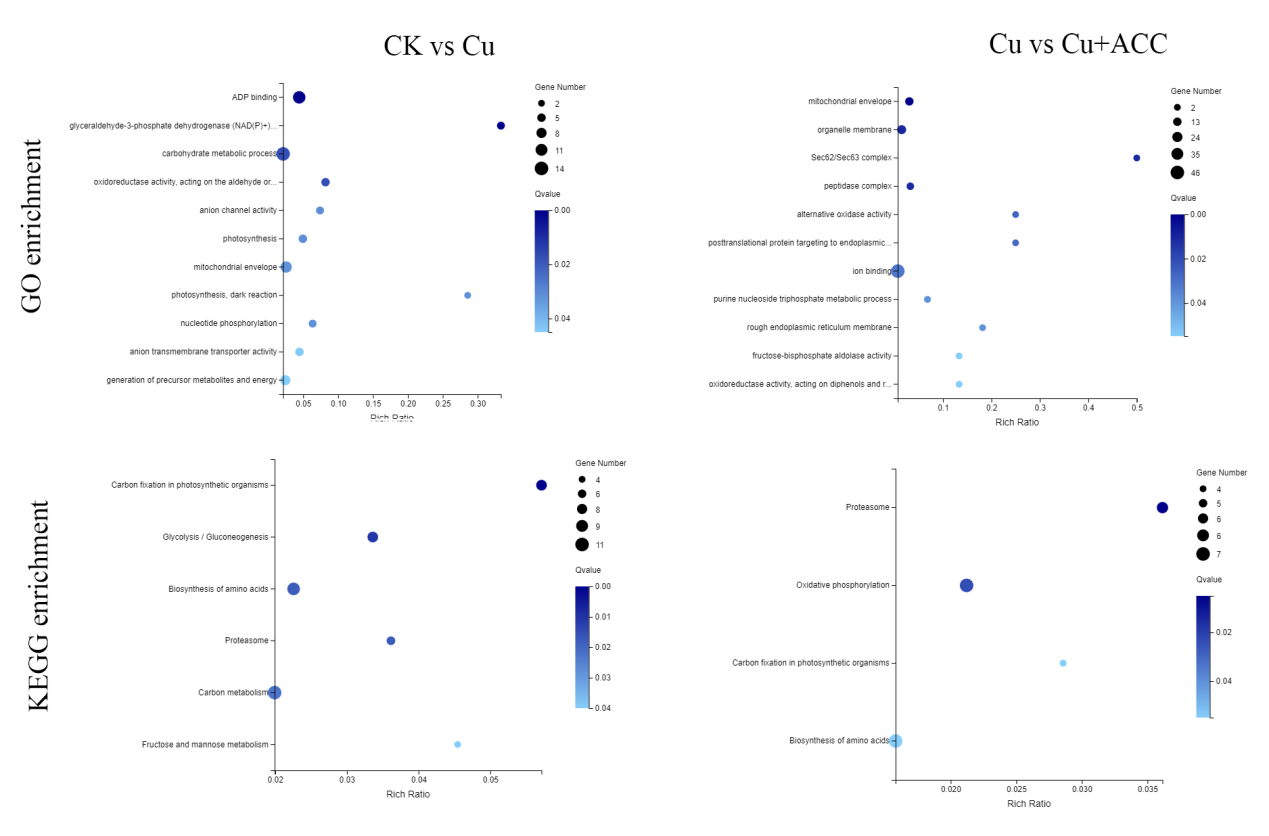


**Figure S5**. The enriched GO categories and KEGG pathways of the transcripts with DAS events under Cu and Cu+ACC conditions.


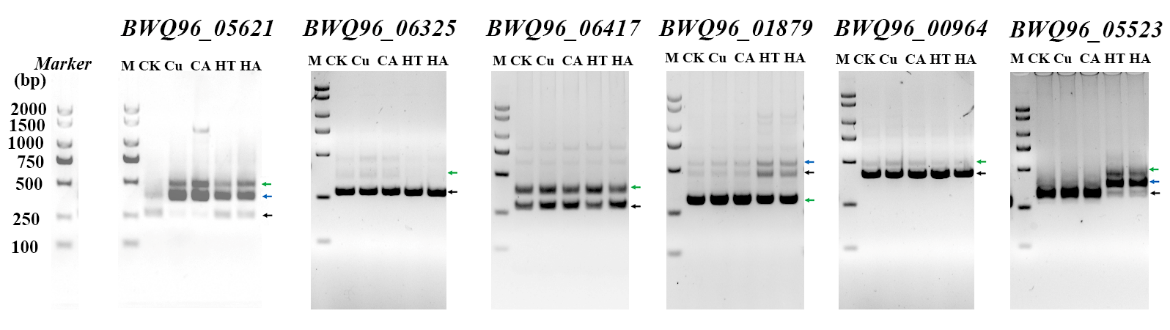


**Figure S6.** Uncropped images PCR of validation of AS events used to prepare Fig 3.


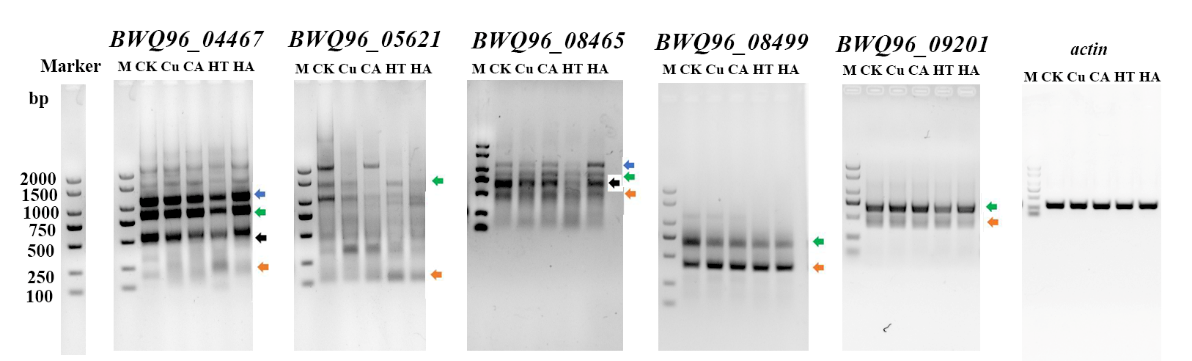


**Figure S7.** Uncropped images PCR of validation of APA events used to prepare Fig 4C.


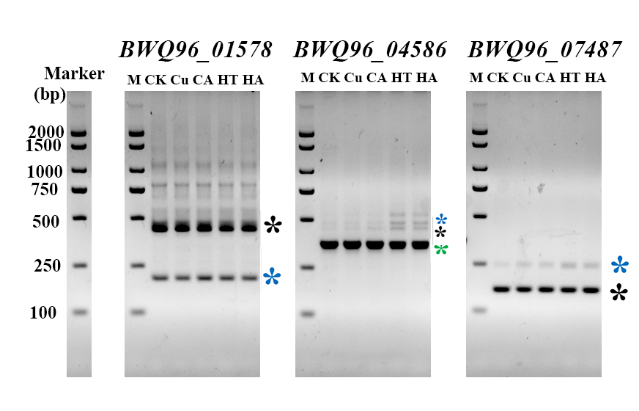


**Figure S8.** Uncropped images PCR of validation of miRNAs used to prepare Fig 5.


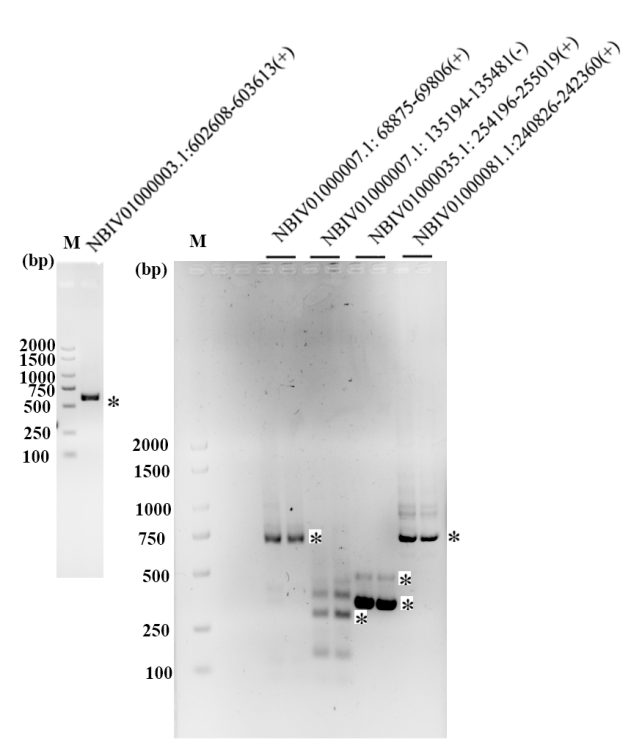


**Figure S9.** Uncropped images PCR of validation of novel genes used to prepare Fig 6.
